# Comprehensive Genomic Analysis of Trihelix Family in Tea Plant (*Camellia sinensis*) and Their Putative Roles in Osmotic Stress

**DOI:** 10.3390/plants13010070

**Published:** 2023-12-25

**Authors:** Zhuoliang Lang, Zelong Xu, Linying Li, Yuqing He, Yao Zhao, Chi Zhang, Gaojie Hong, Xueying Zhang

**Affiliations:** 1 College of Tea Science and Tea Culture, Zhejiang A&F University, Hangzhou 311300, China; 2 State Key Laboratory for Managing Biotic and Chemical Threats to the Quality and Safety of Agro-Products, Institute of Virology and Biotechnology, Zhejiang Academy of Agricultural Sciences, College of Advanced Agricultural Sciences, Zhejiang A&F University, Hangzhou 311300, Chinaanran6297@163.com (L.L.);; 3 College of Advanced Agricultural Sciences, Zhejiang A&F University, Hangzhou 311300, China

**Keywords:** *C. sinensis*, expression profile, phylogenetic analysis, trihelix family (TH), osmotic stress

## Abstract

In plants, Trihelix transcription factors are responsible for regulating growth, development, and reaction to various abiotic stresses. However, their functions in tea plants are not yet fully understood. This study identified a total of 40 complete Trihelix genes in the tea plant genome, which are classified into five clades: GT-1 (5 genes), GT-2 (8 genes), GTγ (2 genes), SH4 (7 genes), and SIP1 (18 genes). The same subfamily exhibits similar gene structures and functional domains. Chromosomal mapping analysis revealed that chromosome 2 has the most significant number of trihelix family members. Promoter analysis identified cis-acting elements in *C. sinensis* trihelix (CsTH), indicating their potential to respond to various phytohormones and stresses. The expression analysis of eight representative CsTH genes from four subfamilies showed that all CsTHs were expressed in more tissues, and three CsTHs were significantly induced under ABA, NaCl, and drought stress. This suggests that CsTHs plays an essential role in tea plant growth, development, and response to osmotic stress. Furthermore, yeast strains have preliminarily proven that CsTH28, CsTH36, and CsTH39 can confer salt and drought tolerance. Our study provides insights into the phylogenetic relationships and functions of the trihelix transcription factors in tea plants. It also presents new candidate genes for stress-tolerance breeding.

## 1. Introduction

Trihelix transcription factors, a plant-specific family of transcription factors, are characterized by their conserved three-helical structure (helix-loop-helix-loop-helix) [1]. These factors are commonly referred to as a GT factor family because they can bind to light-responsive elements (GT elements) [2]. The amino acid sequences of the N-terminal are generally consistent and strongly conserved, but the C-terminal are varied [3]. These transcription factors are generally classified into five subfamilies: GT1, GT2, GTγ, SH4, and SIP1 [1,4]. In contrast to the GT2 subfamilies, which possess two DNA-binding domains (one α-helix domain positioned at the center and the other in the C-terminus), the remaining subfamilies exclusively harbor a single (α-helix domain) in the C-terminal [3].

The first trihelix transcription factor, discovered in peas, was named GT1 [2]. The subsequent characterization of the tobacco GT1 revealed an α-helix domain in the C-terminus [5]. In contrast to GT1, GT2, a trihelix transcription factor in rice, exhibits distinct sequence preferences [6]. The GT1 and SH4 subfamilies’ trihelix domains harbor a tryptophan residue within the interior hydrophobic region of the tandem repeat [7]. Although the SH4 family possesses a more extended domain as compared to the others, the proteins of other subfamilies share an additional α-helix structure downstream of the conserved functional structural domain [8]. The third conserved tryptophan in the GTγ and GT2 subfamilies is replaced by phenylalanine, while it is substituted by isoleucine in the SIP1 subfamily [3,9].

The trihelix family is a small gene family with a relatively modest number of members, ranging from 30 to 60 members in most plant species as compared to other gene families. For instance, *Arabidopsis* possesses 29 members, rice harbors 41 members, poplar possesses 56 members, sorghum contains 40 members, maize has 44 members, chrysanthemum has 20 members, *Oenothera fragrans* has 56 members, and quinoa has 47 members [10,11,12,13,14,15,16,17]. Previous research has demonstrated the crucial role of trihelix transcription factors in plant development. ASIL2 (*Arabidopsis* 6b interacting protein-like2) regulates early embryo development, while ASIL1 exerts negative regulatory control over seedling embryo shape and maintains seed germination under temporal control [18]. *Arabidopsis* PTL (PETAL LOSS) deletion results in fewer petals. NtSIP1 (seed imbibition protein 1) is involved in plant cell proliferation [19]. Tomato SlGT11 plays a role in floral organ identity and the maintenance of floral characteristics [20,21].

Researchers have recently gained a heightened interest in the trihelix family genes due to their critical involvement in abiotic stressors. AtGT4, GhGT26, OsGTγ-1, and OsGTγ-2 enhance plant salt-stress tolerance [22,23,24,25]. The *Arabidopsis* SIP1 subfamily member gene, AtAST1, enhances salt and osmotic tolerances in transgenic plants by regulating stress-responsive genes [26]. AtGTL1 negatively regulates water use efficiency and drought tolerance in *Arabidopsis* by modulating stomatal density [27]. BnSIP1-1 (*Brassica napus*) is induced by ABA (Abscisic acid) and abiotic stress, and it improves seed germination when exposed to osmotic pressure, salt, and ABA treatments [28]. TaGT2L1 negatively regulates drought tolerance and stomatal development in wheat [29]. The overexpression of GmGT-2A, GmGT-2B, Sb06g023980, and Sb06g024110 can enhance *Arabidopsis’s* tolerance to salt, low temperature, and drought stress [4,30]. ZmGT-3b knockdown seedlings exhibit improved drought tolerance [31]. Ptagtl1 regulates stomatal development and plant water absorption in *Populus trichocarpa* [32].

The increasing frequency and severity of high temperatures and drought stress impacts plants’ growth, development, yield, and quality [33,34,35]. Most trihelix genes in *Brassica napus* exhibited significant changes in expression patterns upon heat treatment [36]. Additionally, the expression of 12 SbTHs was significantly upregulated in sorghum in response to high-temperature stress [13]. GT-1 acts as a mediator that links the signal perception and activation of the cellular thermal response by activating the expression of HsfA2 (Heat shock factor A2), a crucial factor for thermotolerance [37]. Persian walnut, a woody economic crop, enhances its tolerance to osmotic stress by regulating stomatal characteristics, maintaining ionic homeostasis, activating stress-responsive genes, and accumulating secondary metabolites [38,39].The tea plant is a globally consumed cash crop renowned for its abundance of secondary metabolites, which contribute to its nutrient content, refreshing taste, and rich flavors that are beneficial to human health [40]. As a woody crop grown and cultivated in both tropical and temperate regions, tea plants have evolved a sophisticated suite of defense mechanisms to mitigate the effects of changing environmental stresses, such as extreme high temperatures and drought [41,42]. However, tea cultivation has expanded into areas with less-than-ideal conditions, leading to excessive salt stress in some regions. Salt stress is an environmental factor that disrupts ion transport and compromises tea plant photosynthesis.

The recently completed genome sequencing of *C. sinensis* has paved the way for unprecedented genome-wide characterization and the analysis of the repertoire of transcription factor families, including those crucial for modulating responses to diverse abiotic stressors. Given the potential roles of the trihelix family genes in stress tolerance, genome-wide analysis of CsTH genes is essential for further research. In this study, we identified a total of 40 potential trihelix genes. We comprehensively analyzed the sequence characteristics, phylogenetic relationships, gene structure, chromosome distribution, and conserved motifs of the CsTH. Additionally, the expression profile of CsTH genes was examined in both the genes’ normal and stressed states in response to salt and drought stress. These findings provide crucial data for further research into the functional genes of CsTH and offer potential gene resources for developing tea plant strains with enhanced stress tolerance.

## 2. Results

### 2.1. Identification and Classification of CsTH Genes in Tea Plants

A total of 40 non-redundant trihelix genes were identified from the genomic and transcriptome database of the tea plant, and their corresponding amino acid sequences matched the ASIL1/2-like (IPR044823) domain and possessed the MYB_DNA-binding domain (IPR044822). All the 40 CsTH genes contained the MYB_DNA-binding domain, and 16 of the genes had the ASIL1/2-like domain. We named these identified TH genes (CsTH1 to CsTH40) according to their location in the chromosome and the number of THs in each chromosome ranging from one (Chr 14) to six (Chr 2). The 40 CsTH proteins ranged from 192 (CsTH24) to 734 (CsTH15) in length with molecular weights (MW) that varied from 21.66 to 82.12 kDa, and isoelectric point (pI) ranges from 4.5 (CsTH10) to 10.54 (CsTH14). In addition, 30 CsTH proteins were predicted to be located in the nucleus, suggesting their function of transcriptional regulation; five were located in chloroplast, two were located in the cytoplasm, and three CsTHs were located in the mitochondrion, Golgi apparatus, and cell wall, respectively (Table S1).

### 2.2. The Phylogenetic Relationship and Protein Structure Analysis of CsTHs

**Figure 1:**
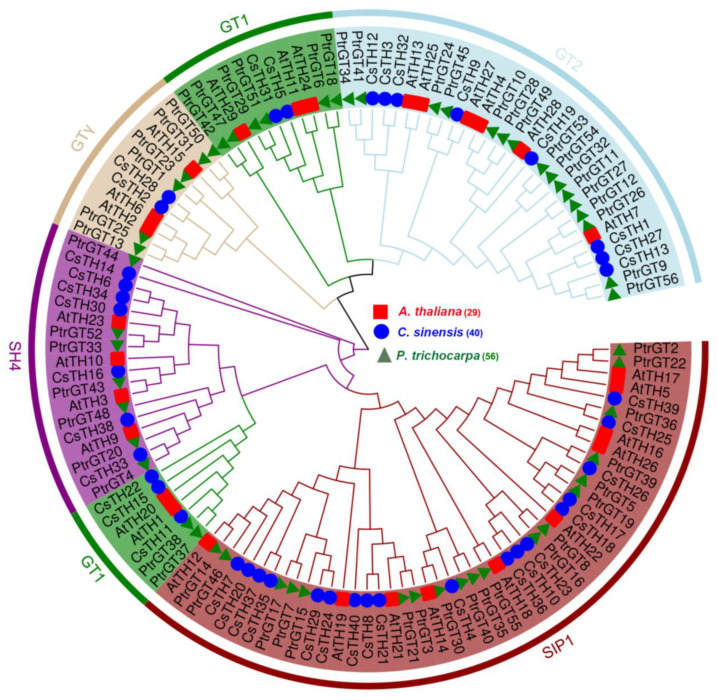
Phylogenetic analysis among 125 trihelix proteins in *Arabidopsis thaliana*, *P. trichocarpa*, and *C. sinensis*. The different colors represent the subfamilies of the trihelix proteins. The trihelix proteins of *Arabidopsis thaliana*, *P. trichocarpa*, and *C. sinensis* are marked with red squares, blue circles, and green triangles, respectively.

**Figure 2:**
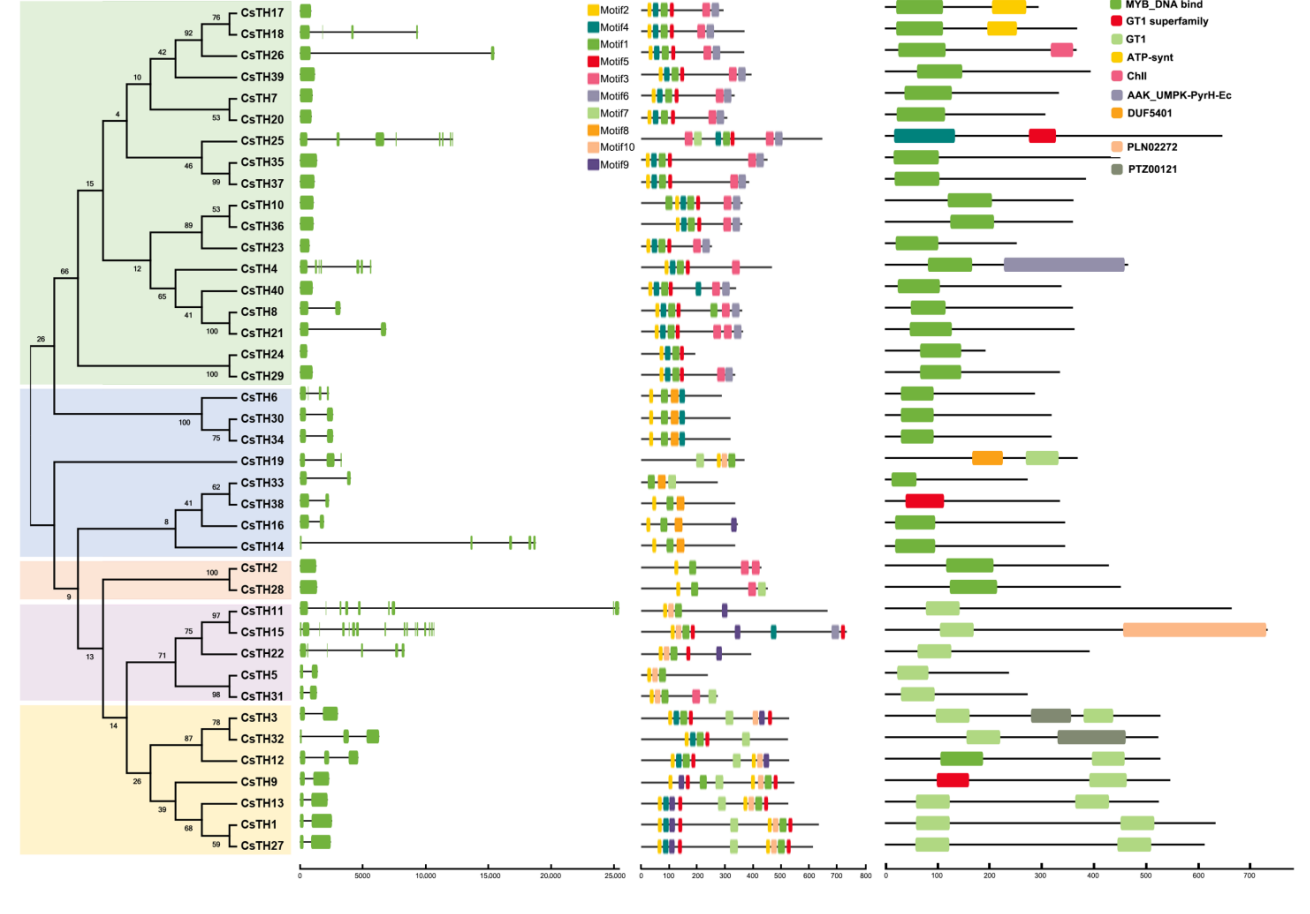
Gene structure, motif compositions, and conserved regions of the trihelix genes in *C. Sinensis*. Exon and intron structures of THs are graphically represented by orange boxes and black lines, respectively. The protein sequences of THs are used to predict the conserved regions and motifs. Conserved motifs are indicated by a colored box numbered 1 to 10.

To explore the phylogenetic relationships of the TH proteins among different species, 126 TH proteins from *C. sinensis*, *Arabidopsis*, and *P. trichocarpa* were used for constructing a phylogenetic tree. The phylogenetic analysis showed that all of these CsTHs could be divided into five classes, with each category containing five in the GT1 subfamily, eight in the GT2 subfamily, two in the GTγ subfamily, seven in the SH4 subfamily, and 18 in SIP1 subfamily, respectively (Figure 1). Exon/intron structure analysis showed that 40 CsTHs contain one to 16 exons. 14 CsTHs lack introns, with 12 in the SIP subfamily and two in the GTγ subfamily. In the GT2 subfamily, five of eight TH genes contained three exons. A motif analysis of the CsTH proteins was obtained with the MEME program, and the results showed that most CsTH proteins had motifs 1, 2, 4, and 5, which correspond to the MYB_DNA-binding domain. Motifs 3 and 6 correspond to the ASIL1/2-like (IPR044823) domain and are mainly presented in the SIP subfamily. Intron/Exon analysis showed that most genes in the SIP1 and GTγ subfamilies only contained one exon, while GT2 and SH4 subfamilies had one intron (Figure 2). Additionally, we found that the genes in the GT1 subfamilies contained more introns than those in the other subfamily. The results indicate that most genes clustered in the identical subfamily showed similar exon/intron structures and arrangements, indicating that the functions of the trihelix proteins in each defined subfamily may be similar.

### 2.3. The Chromosome Distribution and Synteny Analysis of the Trihelix Gene in C. sinensis

To better understand the mechanism of the genomic distribution of CsTHs on *C. sinensis* chromosomes, a chromosome map of CsTHs was constructed based on the genomic sequences of *C. sinensis*. The results showed that all CsTHs were located on the *C. sinensis* chromosomes; Chr 2 contained the most TH members, none of which were on Chr 4 and Chr 7 (Figure S1). We performed further research on 40 CsTHs located on chromosomes and found 15 possible pairs of duplicated genes. According to the results of the gene repetition analysis, all identified paralogous genes were segmental duplications, with no tandem duplication of CsTHs in any chromosomes. This indicates that segmental duplication is the primary expansion mechanism of the CsTH gene family. TB tools calculated Ka/Ks (synonymous/non-synonymous) values, and the ratio of Ka/Ks varied from 0.193 to 0.588, indicating that purifying selection plays a vital role during gene replication (Supplementary Table S2).

**Figure 3:**
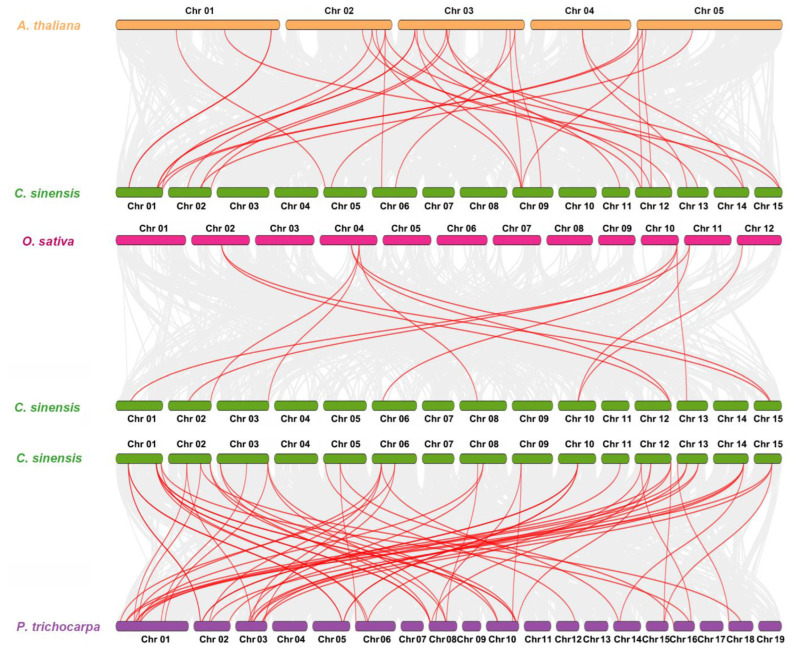
Synteny analysis of TH genes between *C. sinensis* and three representative species. Red lines highlight the orthologous TH gene pairs between *C. sinensis* and other plant genomes, while the gray lines in the background indicate the collinear blocks.

We also investigated the TH homologous gene pairing between different species to explore the species’ evolutionary relationships. Dual synteny analysis revealed that these species also exhibited homology. For example, 20 CsTHs and 19 AtTHs were orthologous gene pairs, resulting in 29 syntenic relationships between *C. sinensis* and *Arabidopsis*. Six OsTHs and 10 CsTHs resulted in 13 syntenic relationships between *Oryza sativa* and *C. sinensis*. Twenty-nine CsTHs and 34 PtTHs were orthologous gene pairs, resulting in 61 syntenic relationships between *C. sinensis* and *P. trichocarpa* (Figure 3). These results propose that TH genes possessed a degree of homology in different species; *C. sinensis* and *P. trichocarpa* exhibited the highest level of homology.

### 2.4. The Promoter Analysis for the CsTH Gene Family

**Figure 4:**
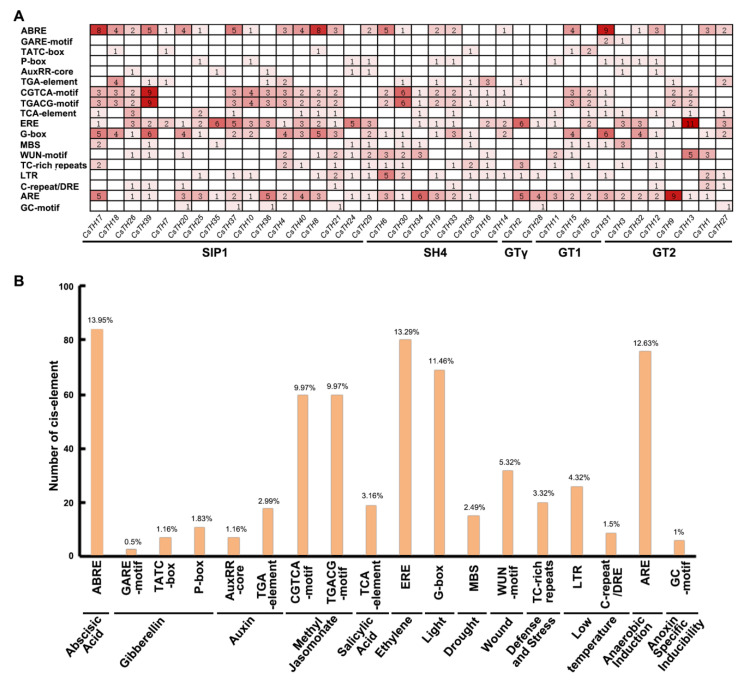
(**A**) These numbers indicate the total number of cis-elements for each CsTH gene. (**B**) The categorization of cis-acting elements relevant to abiotic stresses and plant hormone responses. The percentages and quantities of factors in each category are presented.

In the promoter analysis, two categories of cis-elements were discovered in the 35 CsTH genes. The first category is plant hormone response elements. The number of methyl jasmonate (MeJA)-responsive elements (TGACG-motif/CGTCA-motif) amounts to 120, with an average of three per gene. ABA-responsive elements (ABRE) and ethylene-responsive elements (ERE) come second and third at 84 and 80, with two per gene. By comparison, gibberellin-response elements (GARE, TATC-box, and P-box), Auxin (AuxRR-core and the TGA element), and salicylic acid-responsive elements do not have as many as they do, despite more kinds of motifs. The second category is abiotic and biotic stress-response elements. The anaerobic induction regulatory element (ARE) has the largest number of 76 in total, with an average of 1.9 per gene, and the light-responsive element (G-box), followed by a number of 69. In addition, the number of low temperatures (LTR and C-repeat/DRE), the Wound-responsive element (WUN-motif), defense and stress (TC-rich repeats), as well as drought (MBS)-responsive elements accounted for 35, 32, 20, and 15, respectively (Figure 4A). For the same kind of regulatory elements, it presents preference as codons for THs. The proportion of CGTCA and TGACG element components is similar in Methyl Jasmonate elements. The P-box motif has the largest number as compared to the TATC-box and the GARE-motif for gibberellin-responsive elements. The TGA element has much more than the AuxRR-core element. In low-temperature elements, the LTR element is more than the C-repeat/DRE element (Figure 4B). The detection of these cis-acting elements suggested that the CsTHs can respond to a variety of hormones and stresses, and these response elements may directly affect the stress response ability of CsTHs under stress conditions.

### 2.5. The Tissue and Organ Expression Pattern of CsTHs in the Tea Plant

**Figure 5:**
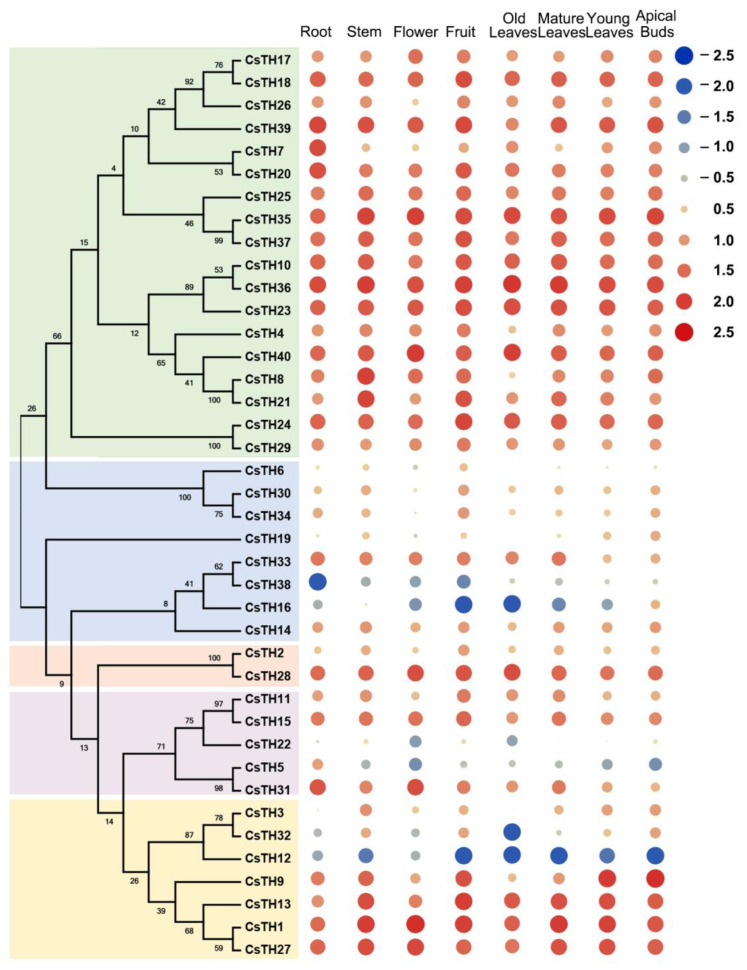
The expression patterns of CsTHs genes in the root, stem, old leaf, flower, fruit, mature leaf, young leaf, and apical bud of the tea plant were calculated using Log_10_^(FPKM)^. Most of the data were distributed between −2.5 and +2.5, the color and size of the dots represent expression levels. Blue dots and red dots are indicated low and high expression levels, respectively.

**Figure 6:**
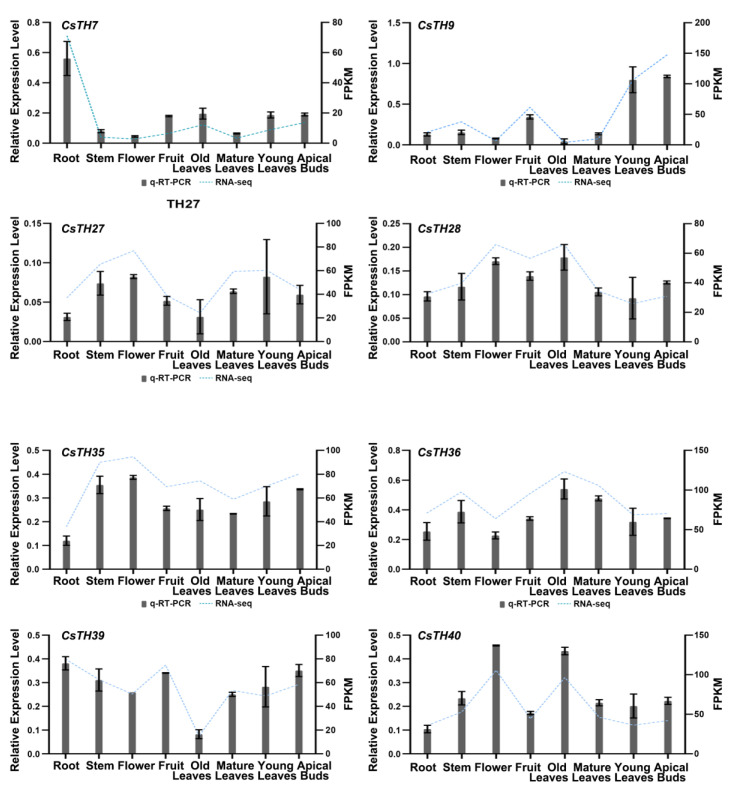
Real-time qRT-PCR analysis of eight TH genes in different tissues in tea plants. Error bars indicate the SD of three biological replicates.

Gene expression patterns can provide helpful information about the physiological functions of CsTHs. The expression profiles covering eight tissues in the tea plant were analyzed using the publicly available RNA-seq dataset released in TPIA to estimate the physiological roles of 40 CsTH genes. The heat map visualized the relative expression of 40 CsTHs and showed a wide range of expressions, and these genes could be grouped into three primary categories. The first expression pattern showed that all CsTHs, except for CsTH33 in the SH4 group, CsTH12, and CsTH22, were extremely low in all of the examined tissues. Compared with these, 14 CsTHs in SIP1, three CsTHs in GT2, and one CsTH gene each in GT1 and GTγ were constitutively expressed with high levels in all the tissues, with some genes displaying high expression levels in specific tissues such as the following: CsTH7 and CsTH20 high expression in the root, and CsTH8 and CsTH21 high expression in the stem. The remaining genes that made up the final expression pattern were also constitutively expressed in all tissues but at low levels (Figure 5). Most CsTHs in the same subfamily displayed similar expression patterns, whereas the CsTHs in different subfamilies were diverse. Based on tissue-specific expression, eight representative CsTHs from four subfamilies were subsequently investigated in other tissues of the tea plant cultivar (Longjing43) utilizing qRT-PCR (quantitative real-time polymerase chain reaction) analyses (Figure 6). The expression patterns of these CsTHs were highly consistent with their transcriptomic profiles from RNA-seq.

### 2.6. The Expression Profiles of CsTHs in Response to Abiotic Stress

**Figure 7:**
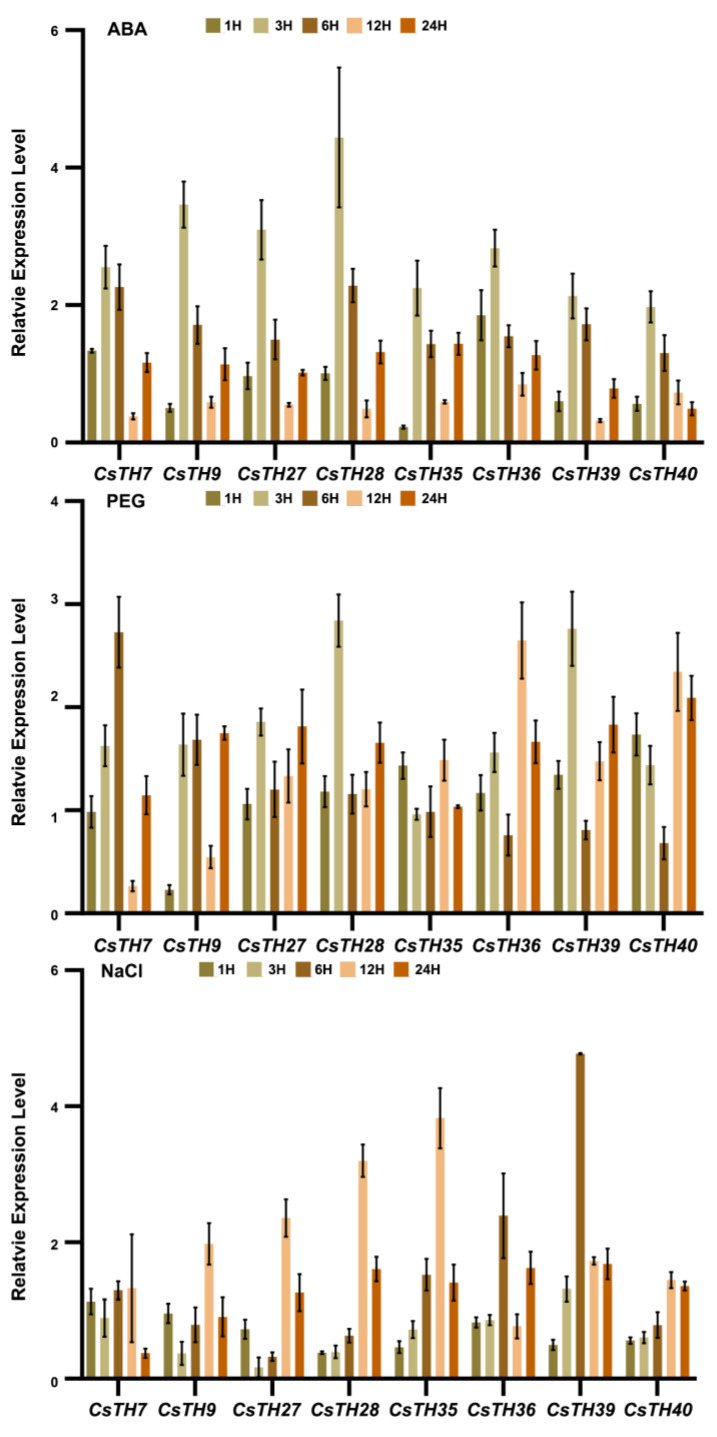
Expression patterns of CsTHs under ABA, PEG, and NaCl stress. Error bars indicate the SD of three biological replicates.

High salt and drought stress accelerate the accumulation of ABA in plants, and the accumulated abscisic acid can induce the expression of ABA-responsive genes and stomatal closure in leaves, improving plant salt resistance and drought resistance [43,44]. Considering the expression of the CsTHs and the existence of ABRE elements in their promoter, we examined the expression profiles of eight CsTHs in response to ABA treatment, drought, and NaCl stress through the quantitative real-time polymerase chain reaction. The results showed that all eight CsTHs were strongly induced in response to ABA treatment and reached peat at 3H. CsTH28 was upregulated more than fourfold. For drought stress, after three h of a PEG-6000 treatment, the expression levels of CsTH28 and CsTH39 were upregulated and gradually decreased. CsTH7 was upregulated and peaked at 6 h, while the CsTH36 and CsTH40 were gradually upregulated and peaked at 12 h. For the NaCl treatment, the expression levels of CsTH9 and CsTH27 were decreased and then gradually increased to high levels at 12h. CsTH28 and CsTH35 expression levels remained the same at the beginning. Then, they upregulated to high levels at 12 h; CsTH36 and CsTH39 were upregulated and peaked at six h (Figure 7). The integration of expression data under multiple treatments suggested that CsTH28, CsTH36, and CsTH39 responded to drought and salt stress, and thus, we further investigated its function.

### 2.7. The Subcellular Localization and Transcriptional Activation Activities of CsTH28/36/39

**Figure 8:**
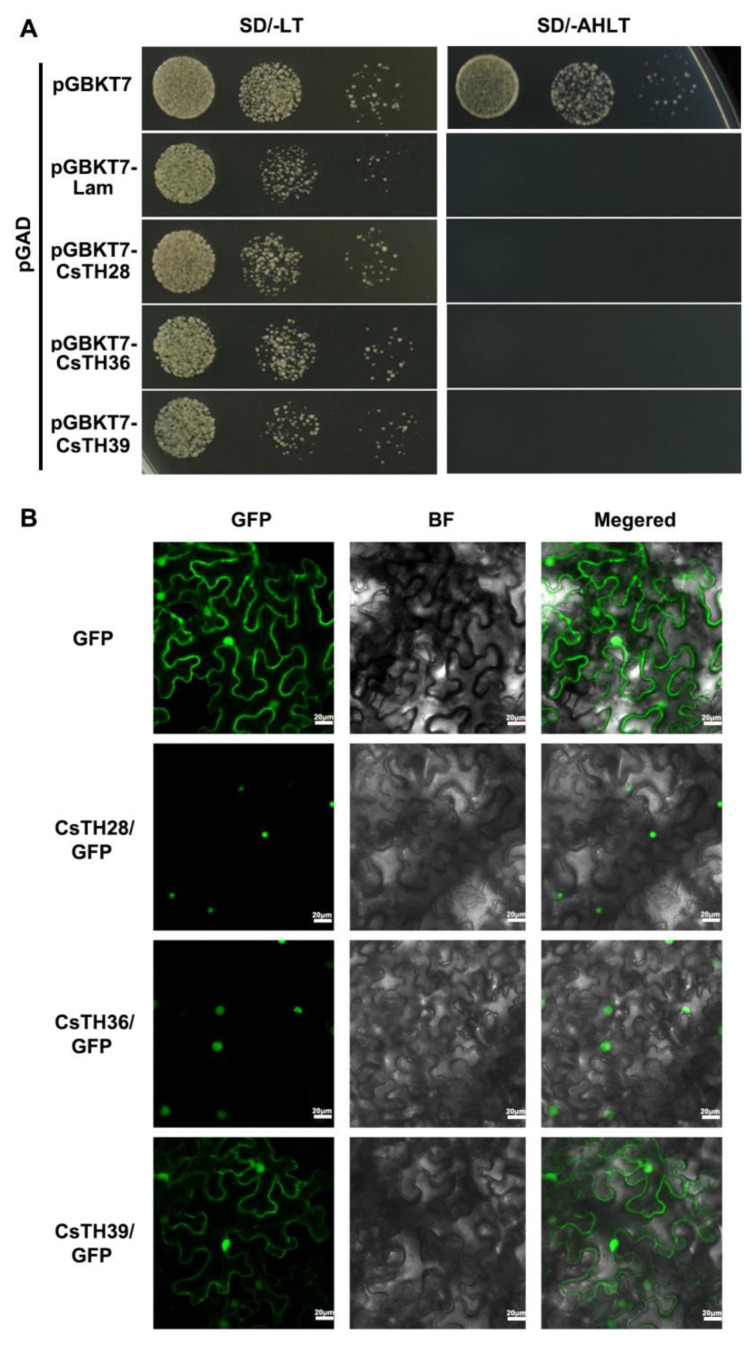
The potential function of CsTHs in tea plants. (**A**) Transactivation analyses of CsTH28, CsTH36, and CsTH39 in yeast. Negative control (pGBKT7-Lam) and positive control (pGBKT7-53) were transformed into the AH109 strain and successively incubated in SD/-Leu/-Trp media and an SD/-Ade/Trp/-Leu/-Trp/-His plate. (**B**) Subcellular localization of CsTH28, CsTH36, and CsTH39. GFP, CsTH28-GFP, CsTH36-GFP, and CsTH39-GFP were transiently expressed in tobacco leaves. GFP: Green fluorescence image, BF: Bright-field microscopy image, Merge: Merged bright-field and green fluorescence images.

The expression levels of the CsTH28/36/39 genes were relatively high with the ABA, drought, and NaCl treatments. The constructed GFP: Pcambia1300-TH28/36/39 fusion vectors and the 1300 empty vector were independently transiently transformed into tobacco (*Nicotiana benthamiana*) leaves. The protein-coding nucleotide products of CsTH28 and CsTH36 were mainly expressed in the nuclei, while CsTH39 was localized at the nuclei and cell membrane (Figure 8A).

The transcriptional activation of CsTH28/36/39 was investigated by constructing pGBK vectors that were then transformed into the yeast strain AH109. The results of transcriptional activation activity showed that CsTH28/36/39 could grow on the SD/-Leu/-Trp medium as well as negative (pGBKT7-lam) and positive (pGBKT7-53) controls. In contrast, the yeast strains transformed with the negative control pGBKT7-lam vector and CsTH28/36/39 could not grow on the SD/-Ade/-His/-Leu/Trp medium. Only the transformants containing pGBKT7-53 can grow on an SD-Dropout-deficient medium, indicating that the full-length sequences of CsTH28/36/39 did not possess transcriptional activation activity to activate the expression of downstream reporter genes (Figure 8B).

### 2.8. The Function of CsTH28/36/39 in Responsive to Osmotic Stress

**Figure 9:**
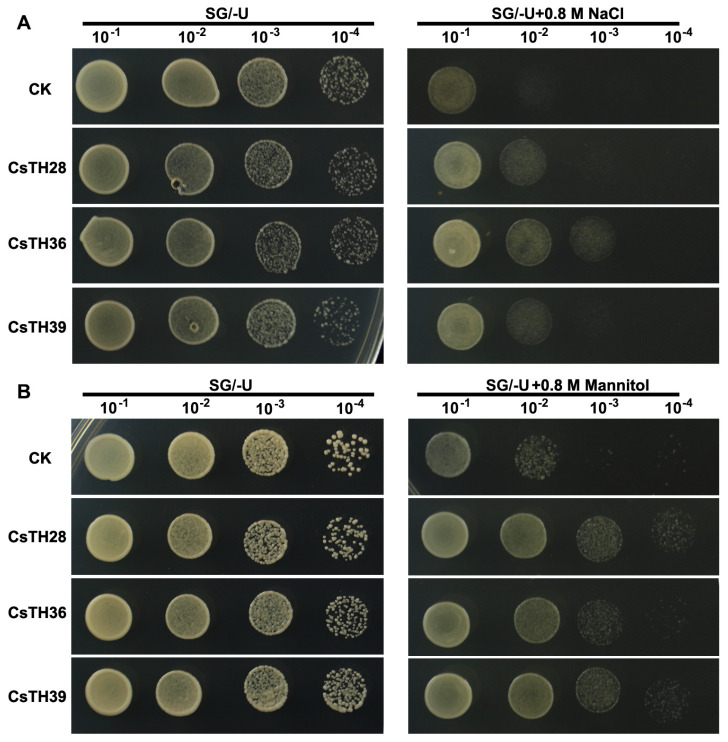
Overexpression of CsTHs improves osmostress tolerance in transgenic yeast. (**A**) The empty vector (CK) and the CsTHs spotted onto SG-U plates with and without 0.8 M NaCl and grown at 30 °C for 3–5 days. (**B**) The empty vector and the CsTHs were spotted onto SG-U plates with and without 0.8 M mannitol and grown at 30 °C for 3–5 days.

Transcription is crucial in long-term stress adaptation and protection against future stresses. Owing to efficient validation, yeast has enabled studies of gene regulation and biology under stress. Thus, to assess the relevance of the CsTH28/36/39 on cell growth upon osmotic, we transformed the CsTH28/36/39 into the *S. cerevisiae* (INVSc1 and BY4741) strain and monitored cellular growth in the presence of high osmolarity (0.8 M NaCl and 0.8 M mannitol). The growth of the control (INVSc1 and BY4741 transformed with empty vector) and recombinant strain containing CsTH28/36/39 maintained growth on the SG/-U medium. However, wild-type cells impaired growth under high osmolarity conditions (0.8 M NaCl and 0.8 M mannitol). Under salt treatment, the yeast cells overexpressing CsTH28 and CsTH39 could grow properly when the cells were diluted by 10^2^, while CsTH36 could grow normally when the cells were diluted by 10^3^. Under mannitol treatment, the yeast cells overexpressing CsTH28 and CsTH39 could grow properly when the cells were diluted by 10^4^, while CsTH36 could grow normally when the cells were diluted by 10^3^. These results show that CsTH28/36/39 could enhance the tolerance to osmotic stress, with CsTH36 conferring the highest tolerance to salt stress, while CsTH28 and CsTH39 show higher tolerance to mannitol than CsTH36 (Figure 9). These results indicated that the CsTH28/36/39 gene was involved in response to salt and drought stress in tea plants.

## 3. Discussion

Early research revealed that the GT factor family regulates morphogenetic processes and acts as a class of light regulators, which explains their roles in controlling light-responsive genes [45]. However, more recent studies have provided compelling evidence that the trihelix family also plays a significant role in plant growth and development and the responsiveness to environmental cues [27,46]. In this study, we identified 40 trihelix genes in the *C. sinensis* genome, distributed across chromosomes with varying densities. Previously, trihelix family genes were classified into three distinct subfamilies (GTα, GTβ, and GTγ) [22]. However, Kaplan-Levy et al. (2012) classified trihelix genes from *Oryza sativa* and *Arabidopsis* into five clades: GT-1, GT-2, SH4, SIP1, and GTγ. Recently, a new subfamily, GTδ, was identified in tomato and rice [11,47]. Phylogenetic analysis demonstrated that 40 CsTHs were divided into five subfamilies, with subfamily members sharing similar gene structures and motif distributions, implying that these genes may have shared a common ancestor and similar functions. Furthermore, at least one CsTH protein was detected in each subgroup of AtTH and PtTH proteins, indicating that the differentiation of the trihelix TF family occurred prior to the divergence between monocots and dicots.

The similarity in gene structures and motif distributions among subfamily members suggests that they may have originated from gene duplication events. Fifteen segment duplication events were identified among 40 CsTHs, which supports this hypothesis. Furthermore, a group of CsTHs was linked to numerous syntenic gene pairs, suggesting that these genes may play a significant role in the evolution of the trihelix gene family and were essential for plant development and defense mechanisms. All segmental duplication events in the *C. sinensis* gene trihelix family occurred during a recent whole genome duplication event. The duplicated genes that experience sub-functionalization through purifying selective pressure (Ka/Ks < 1), neo-functionalization through positive selective pressure (Ka/Ks > 1), and non-functionalization play essential roles in adaptive evolution. In this study, all of the Ka/Ks ratios in the different trihelix gene pairs are less than one, suggesting that all the duplicated genes experience a strong purifying selective pressure and are sub-functionalized during evolution. This phenomenon is consistent with the fact that most duplicated genes are sub-functionalized in *C. sinensis*. Additionally, gene synteny analyses showed that most CsTH genes displayed syntenic relationships with those in *Arabidopsis* and *populus*. Conversely, there are fewer collinearity gene pairs with monocotyledonous plants, with only 13 gene pairs with rice. This suggests that some redundant TH genes were lost during evolution.

Light plays a crucial role in influencing tea leaf quality. Early research focused on the role of GT factors in regulating gene expression in response to light [48,49]. However, subsequent studies revealed that these genes also contribute to plants’ responses to salt, drought, and hormone signaling [11,50]. The cotton gene Gh_A05G2067 shows increased expression under drought and salt stress [51]. Similarly, the tomato GT-1 gene ShCIGT displays increased expression under drought and low-temperature stress [20]. Upstream regulatory factors can regulate the expression levels of target genes by recognizing and binding to cis-acting elements located on the promoter region. These regulatory elements, in turn, govern various biological processes. Over the years, the functions and pathways associated with many cis-acting elements have been deciphered. An analysis of the promoter sequences of TH genes in *C. sinensis* revealed a significant abundance of hormone- and stress-responsive elements. Plant hormones are organic compounds produced in trace amounts within plants, and they play a crucial role in regulating various physiological processes throughout their life cycle. Abscisic acid (ABA), commonly referred to as a stress hormone, regulates stomatal opening, growth, and development, but also contributes to plant responses to abiotic stress [52,53]. By conducting qRT-PCR, we observed that all of the selected genes were regulated by ABA treatment. Our focus on the expression of the eight TH genes mentioned above revealed that *CsTH28*, *CsTH36*, and *CsTH39* exhibited distinct responses to drought and salt treatment. These three CsTH genes could be promising candidates for further investigations into abiotic stress tolerance in *C. sinensis*.

Because transcription factors have nuclear localization signal (NLS) regions, most transcription factors are localized within the nucleus. However, there are exceptions such as AtHSFA6a, which resides in the cytoplasm, and TaMIF4-5D, which is found both in the nucleus and cell membrane [54,55]. We identified CsTH28 and CsTH29 as nuclear-localized genes. This observation suggests that these two genes may function as transcription factors. However, some transcription factors exhibit dual localization, meaning that they are present both in the nucleus and the cell membrane. Previous studies have demonstrated the intricate roles of transcription factors in plant stress responses. Under salt stress, the NAC (NAM, ATAF and CUC) transcription factor AtNTL8 translocates from the cytoplasm to the nucleus, activating downstream stress resistance genes and enhancing plant tolerance [56,57]. Similarly, the TaNTL1 transcription factor rapidly shuttles between the plasma membrane and the nucleus under drought stress, relaying signals to activate early response genes [58]. AtNLT8 and TaNTL1 contain transmembrane motifs, and both are released from the membranes by proteolytic cleavage, possibly in response to stress conditions, and transported into the nucleus where they regulate the expression of stress responsive genes. Comparatively, drought treatment triggers the expression of the OsFTIP6-OsHB22-OsMYBR57 module. OsFTIP6 (FT-INTERACTING PROTEIN6) interacts with OsHB22, promoting its nucleocytoplasmic shuttling, where it further interacts with OsMYBR57 to directly activate OsbZIPs (basic leucine zipper), enhancing drought tolerance in rice [59]. We performed transmembrane domain prediction analysis on CsTH39 and found that it does not contain transmembrane domain. Our findings regarding CsTH39’s location in both the nucleus and cell membrane and its precise localization and response to osmotic stress warrant further in-depth investigation. The transcriptional auto-activation experiment using yeast two-hybrid assay revealed that none of the CsTH genes exhibited auto-activation activity. It is speculated that the proteins of the CsTH family may regulate gene expression or participate in different physiological functions by forming homotypic or heterodimers.

Drought and salinity, as major environmental abiotic stresses, negatively impact crop development, yield, and quality. It is well-known that osmotic stress response mechanisms involve several particular physiological and biochemical pathways that allow Persian walnut to adapt to unfavorable conditions [60].The regulatory roles of certain TH genes in salt and drought responses have been reported. In soybean plants, the transgenic GmGT-2B and GmGT2A plants enhance salt tolerance, frost resistance, and drought resistance, and they respond to ABA [30]. Trihelix transcription factors play a role in *Arabidopsis* by influencing embryonic development and the plant’s response to drought and salt stress [61,62]. Among the multitude of genes involved in various abiotic stress responses in rice, OsGTγ-1, and SHA1 stand out as compelling examples: overexpression of OsGTγ-1 bolsters salt stress tolerance in rice seedlings, whereas mutations in SHA1 lead to delayed abscission of rice seeds [11,22,63]. The overexpression of the wheat trihelix gene TaGT2L1D in *Arabidopsis* increased the number of stomata in *Arabidopsis* leaves and decreased the drought tolerance of the plants [29]. Under abiotic stress conditions, yeast will initiate stress responses and regulate gene expression to enhance stress resistance. At present, many studies have performed antistress gene phenotypic validations through yeast due to the fact that the validation procedure is simple, efficient, and has wide applications [64,65,66]. We made a preliminary exploration of the function of CsTH genes in response to osmotic stress in yeast. Consistent with findings in other species, the overexpression of CsTH28/36/39 in yeast strains increased tolerance to osmotic stress. These data suggest that CsTH28/36/39 might play a crucial role in osmotic stress response and pave the way for further investigations.

## 4. Materials and Methods

### 4.1. Identification of CsTH genes in the C. sinensis Genome

The previously reported TH-encoded proteins from *Arabidopsis* [67] and *P. trichocarpa* [12] have close phylogenetic relationships with the tea plant. Genewise v2.2.0 software [68] was used to search the TH sequence from the tea plant genome sequence using amino acid sequences from *Arabidopsis* and *P. trichocarpa*. The Pfam database (http://pfam.sanger.ac.uk, accessed on 1 September 2023) was used to analyze the Hidden Markov model (HMM) of the selected CsTHs. SMART (http://smart.embl-heidelberg.de/, accessed on 3 September 2023) and the NCBI Conserved Domain Database (http://www.ncbi.nlm.nih.gov/Structure/cdd/wrpsb.cgi, accessed on 4 September 2023) were used to filter the redundant sequences and confirmed all the potential CsTH genes (for the existence and integrity of the TH domain and domain). ExPASy was used to predict the isoelectric point (pI), molecular weight (MW), and grand average of hydropathicity (GRAVY). WoLF PSORT was used to predict the subcellular localization of the CsTH proteins.

### 4.2. Conserved Motifs, Gene Structure, Promoters, and Evolutionary Analyses of the THs

The TH protein sequences from *Arabidopsis*, *P. trichocarpa*, and *C. sinensis* were aligned using the ClustalX 1.83 program. Phylogenetic trees for three species of TH protein sequences were constructed by MEGA 6.0 software using the NJ (Neighbor-Joining) method with the following parameters: the bootstrap method (1000 replicates), the Poisson model, uniform rates, and complete deletion. The motif of CsTHs was conducted on the MEME website (http://meme-suite.org/tools/meme, accessed on 5 September 2023) with the maximum number of motifs set as 10. The intron and exon were displayed by the online website gene structure display server (GSDS 2.0, http://gsds.cbi.pku.edu.cn, accessed on 5 September 2023). Cis-acting regulatory elements were annotated using an online tool (http://bioinformatics.psb.ugent.be/webtools/plantcare/html/, accessed on 8 September 2023). The basic characteristic data about the TH proteins were calculated using the program tool (http://web.expasy.org/protparam/, accessed on 8 September 2023). The secondary structures of the proteins were analyzed using a scratch protein predictor (http://scratch.proteomics.ics.uci.edu/, accessed on 10 September 2023).

### 4.3. Analyses of Synteny

The CsTH genes’ physical position in the *C. sinensis* genome database was established by Tbtools. The CsTH gene duplication events were examined by MCScanX software (University of Georgia, Athens, GA, USA) with default parameters. The matching genes were drawn by the Circos-0.67 program to visualize the duplicated regions in the *C. sinensis* genome (http://circos.ca/, accessed on 11 September 2023). The homology of the TH genes between *C. sinensis* and the other species (*Arabidopsis*, *O. sativa*, *P. trichocarpa*) was analyzed by a dual synteny plotter of Tbtools [69].

### 4.4. TH Transcription Factors Expression Pattern in C. sinensis

The expression pattern data of eight tissues and four kinds of abiotic stress, including root, stem, old leaf, mature leaf, young leaf, apical bud, flower, and fruit, as well as cold, PEG, MeJA, and NaCl stress treatments, were downloaded from the Tea Plant Information Archive (TPIA). The expression level of the TH genes was displayed as Log10 FPKM (fragments per kilobase of exon per million fragments value) in a heat map using the Mev4.9.0 software. The ABA and PEG treatments were documented according to a previous report [41]. An amount of 100μM ABA was used to spray the tea plant leaves for ABA treatments (ddH_2_O as control). For drought treatments, 20% PEG was rinsed into the tea plant roots (ddH_2_O as mock control). The primers for CsTHs were designed by Beacon Designer 7.0 software. Glyceraldehyde-3-phosphate dehydrogenase (GAPDH, accession number: KA295375.1) was used as an internal reference. The real-time PCR on an ABI7900HT sequence detection system was found by using SYBR Green (Applied Biosystems, Waltham, MA, USA).

### 4.5. RNA Extraction and qRT-PCR Analysis

According to the manufacturer’s instructions, the total RNA was extracted from plant materials using an RNAprep Pure Plant Kit. The first-strand cDNA was synthesized from the total RNA using a PrimeScript RT reagent kit according to the manufacturer’s instructions–the program and reaction system of qRT-PCR as per the previously reported protocol. The *CsGAPDH* gene was selected as the internal control. All specific primers are listed in Table S3. In different cases, the relative gene expression was quantified using 2^−ΔCT^ methods.

### 4.6. Transcriptional Self-Activating of CsTHs Proteins

The pGBK-CsTHs and pGBK constructs were transformed into the yeast strain AH109, and the transformants were inoculated on an SD/-Trp medium. Further, the colonies were transferred to an SD/-His/-Leu/-Trp medium and incubated at 30 °C for 3–5 days.

### 4.7. Subcellular Localization of CsTHs Proteins

The CsTHs without stop codons were amplified using primers with homologous arms and appropriate enzyme digestion sites (BamH I) before being cloned into the pCambia1300-35S-EGFP vector using the 2× Ezmax^®^ Universal CloneMix (Tolo Biotech., Shanghai, China). This allowed the CsTH genes to fuse with the GFP protein driven by the 35S promoter when expressed in tobacco (*N. benthamiana*) leaves. Two days after infiltration, the expression of GFP in tobacco leaves was examined using a Leica TCS SP5 confocal laser scanning microscope (Leica Microsystems, Bannockburn, IL, USA) 44–48 h after infiltration.

### 4.8. Validation of Gene Function in Yeast

pYES2 is a high-copy-number plasmid vector that leverages the potent, galactose-inducible GAL promoter system to allow tightly regulated, high-level gene and protein expressions in yeast. It contains supporting elements like the ura3 marker for transformant selection [70]. The full-length coding sequences of CsTHs were amplified by PCR to generate pYES2-CsTHs yeast expression vectors using the 2× Ezmax^®^ Universal CloneMix (Tolo Biotech.). The pYES2 vector was linearized by digestion, using the restriction enzymes KpnI and BamHI. INVSc1 and BY4741 are well-suited hosts for heterologous protein expression due to their capacity to carry out post-translational modifications on translated proteins [66,71]. pYES2 and pYES2-CsTHs plasmids were transformed into INVSc1 and BY4741 by the lithium acetate method. These yeasts were plated on an SD/-U medium and validated by PCR after three days of incubation at 30 °C. Subsequently, the transformed INVSC1 yeast and BY4741 yeast were spotted onto the SG/-U medium with 0.8 M NaCl and 0.8 M mannitol, respectively.

### 4.9. Statistical Analysis

All experiments were carried out with at least three independent biological replicates. Each measurement was carried out in triplicate. Data represent the mean  ±  sd of three biological replicates.

## 5. Conclusions

In conclusion, 40 members of the trihelix gene family were identified in the *C. sinensis* genome and were classified into five subfamilies based on phylogenetic relationships. Members of the same trihelix gene subfamilies share similar gene structures and conserved functional domains. All the identified segmentally duplicated gene pairs underwent strong purifying selections over evolutionary timescales. The differential expression profiles of trihelix genes under high salt stress and PEG treatments revealed three candidate genes for further investigating the stress tolerance mechanisms in *C. sinensis*. In conclusion, the present study furnishes a solid foundation for future investigations to elucidate the intricate functions and molecular mechanisms of trihelix genes, advancing our understanding of their roles in plant growth, development, and stress tolerance.

## Data Availability

All datasets presented in this study are included in the article/Supplementary Tables.

## Supplementary Materials

The following supporting information can be downloaded at: https://www.mdpi.com/article/10.3390/plants13010070/s1, Figure S1: Distribution of 40 CsTH genes on 15 chromosomes.; Table S1: Genome-wide bioinformatics analysis of Trihelix genes in *C. sinensis*; Table S2: Ka/Ks value for duplicate trihelix genes in *C. sinensis*; Table S3: Oligonucleotide primer sequences.
