# Supplementary figures and images for: Comprehensive Genomic Analysis of Trihelix Family in Tea Plant (Camellia sinensis) and Their Putative Roles in Osmotic Stress
Source: Plants (Basel). 2023 Dec 25;13(1):70. doi: 10.3390/plants13010070 (PMC10780335; doi:10.3390/plants13010070)

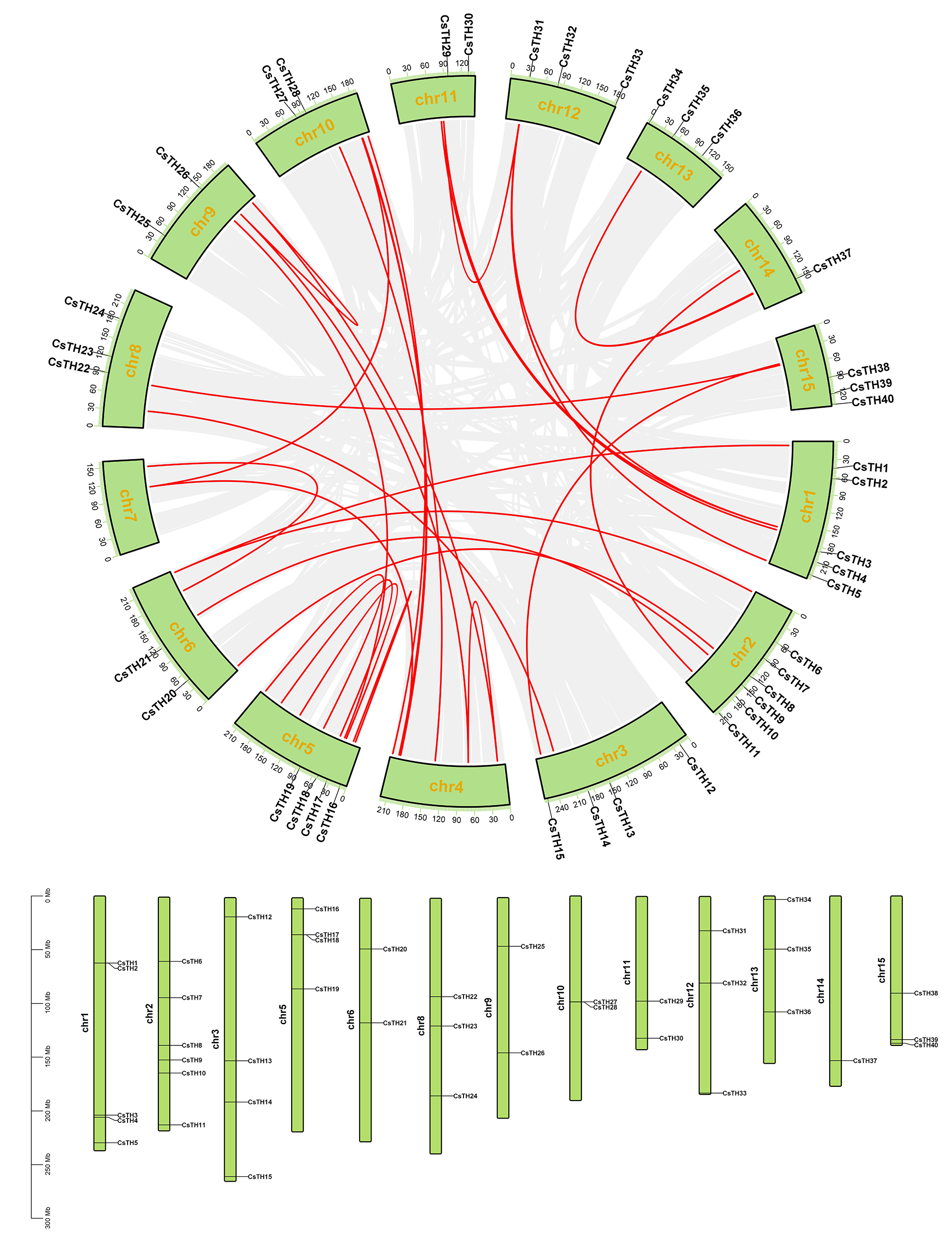

Supplement: Supplementary file 1 [file plants-13-00070-s001.zip › Fiugre S1.tif]
